# Attenuated Expression of *DFFB *is a Hallmark of Oligodendrogliomas with 1p-Allelic Loss

**DOI:** 10.1186/1476-4598-4-35

**Published:** 2005-09-12

**Authors:** J Matthew McDonald, Valerie Dunmire, Ellen Taylor, Raymond Sawaya, Janet Bruner, Gregory N Fuller, Kenneth Aldape, Wei Zhang

**Affiliations:** 1 Departments of Pathology and Neurosurgery, the University of Texas M. D. Anderson Cancer Center, Houston, Texas, USA

## Abstract

Allelic loss of chromosome 1p is frequently observed in oligodendroglioma. We screened 177 oligodendroglial tumors for 1p deletions and found 6 tumors with localized 1p36 deletions. Several apoptosis regulation genes have been mapped to this region, including *Tumor Protein 73 (p73)*, *DNA Fragmentation Factor subunits alpha *(*DFFA*) and *beta *(*DFFB*), and *Tumor Necrosis Factor Receptor Superfamily Members 9 *and *25 *(*TNFRSF9, TNFRSF25*). We compared expression levels of these 5 genes in pairs of 1p-loss and 1p-intact tumors using quantitative reverse-transcriptase PCR (QRTPCR) to test if 1p deletions had an effect on expression. Only the *DFFB *gene demonstrated decreased expression in all tumor pairs tested. Mutational analysis did not reveal *DFFB *mutations in 12 tested samples. However, it is possible that *DFFB *haploinsufficiency from 1p allelic loss is a contributing factor in oligodendroglioma development.

## Introduction

Oligodendroglial tumors with allelic losses on 1p usually display loss of relatively long regions, a phenomenon that has made the identification of putative 1p tumor suppressor genes difficult [1-6]. However, the vast majority of reported oligodendroglioma cases with 1p-loss have involved the 1p36 region, with several breakpoints within the region observed [5-9]. It is important to note that several apoptotic genes have been mapped to 1p36. Diminished apoptosis has been recognized as one of the hallmarks of most types of cancer, representing one of the major ways known for a tumor-cell population to expand [10]. Therefore, we tested *TP73, TNFRSF9, TNFRSF25, DFFA*, and *DFFB*, all of which are 1p genes involved in apoptosis, for differential expression in 1p-status subsets of oligodendroglioma. QRTPCR analysis of match-paired samples demonstrated that levels of *DFFB *were decreased in all 1p-allelic loss cases. In contrast, the other tested genes showed heterogeneous patterns of expression. This result suggests *DFFB *to be a key molecule affected by 1p-deletion in oligodendroglioma.

## Materials and methods

### Samples

The records of 177 patients who underwent treatment for oligodendroglial tumors at the University of Texas M.D. Anderson Cancer Center (UTMDACC) between 1981 and 2002 were collected and reviewed. These patients were initially diagnosed as having low-grade oligodendroglioma or mixed oligoastrocytoma, anaplastic oligodendroglioma or mixed oligoastrocytoma, or glioblastoma multiforme with significant oligodendroglial component by neuropathologists from UTMDACC and later confirmed by two of the authors (KA and GF). Mixed tumors were included in this study since clear pathologic discrimination between glioma subtypes is sometimes difficult, and as a group, oligoastrocytomas often have 1p deletions [4]. In fact, both the oligodendroglial and astrocytic components of mixed tumors have been observed to have this genetic signature [4].

Tissue for DNA isolation was obtained from paraffin-embedded samples. Each tissue block was histologically assessed for tumor by a neuropathologist (KA). Sections were directly cut from the block for DNA isolation if at least 90% of the tissue was determined to be tumor. If the proportion of tumor was <90%, 10 to 20 unstained slides were prepared from the block and tumor tissue was dissected from normal tissue. DNA was isolated by digesting deparaffinized tumor sections for 3 to 5 days with proteinase K at 55°C (0.5 mg/ml in 100 mmol/L NaCl, 10 mmol/L Tris-HCl, pH 8.0, 25 mmol/L ethylenediaminetetraacetic acid, 0.5% sodium dodecyl sulfate), followed by a phenol:chloroform:isoamyl alcohol extraction and isopropanol precipitation.

Tissue for RNA isolation was obtained from fresh/frozen samples. Each frozen section was histologically assessed for tumor by a neuropathologist (GF or KA) and used only if at least 90% of the tissue was determined to be tumor. For RNA isolation, up to 50 mg of tissue was frozen in liquid nitrogen, crushed into powder using a mortar and pestle, and dissolved in 1 ml of Trizol^® ^Reagent. 200 μl chloroform was added to the sample, vortexed at high speed for 15 seconds, and centrifuged at 12,000 × g for 15 minutes at 4°C. After transfer of the aqueous phase to fresh 1.5 ml Eppendorf tube, an equal volume of 70% ethanol was added and mixed by tube inversion. The sample was then loaded onto a QIAGEN RNeasy^® ^mini column and centrifuged at 16,000 × g for 20 seconds (QIAGEN Sciences, Germantown, MD). The column was washed twice with 500 μl of QIAGEN's RPE buffer. RNA was eluated off the column in 50 μl of nuclease-free water. RNA was quantified using a spectrophotometer and qualitated with an Agilent BioAnalyzer 2100 (Agilent Technologies, Palo Alto, CA).

### Detection of 1p allelic loss

Quantitative Microsatellite Analysis (QuMA) was used as previously described to determine 1p allelic loss in 177 tumors in this study [11-13].

### Quantitative Reverse Transcription-Polymerase Chain Reaction (QRTPCR) Analysis

Initial experiments were performed to determine the valid range of RNA concentrations and to demonstrate the similarity of PCR efficiencies for each gene of interest compared to the endogenous control gene cyclophilin. To determine fold-changes in each gene, QRTPCR was performed on the ABI Prism 7700 using the commercially available gene expression assay for *p73, DFFA*, and *DFFB *(Hs00232088_m1, Hs00189336_m1, Hs00237077_m1, respectively) and the cylophilin Vic-labeled Pre-Developed Assay Reagent (Applied Biosystems, Foster City, CA) without multiplexing. In triplicate, we amplified 50 ng cDNA for each sample for each assay in a reaction containing 1× TaqMan^® ^Universal PCR Master Mix without AmpErase UNG and 1× gene expression assay with the following cycling conditions: 10 minutes at 95°C, then 45 cycles of 95°C for 15 seconds and 60°C for 1 minute. Calculations were performed using the δδCt method to determine fold-difference in 1p-loss cases relative to the matched 1p-intact cases. Fold changes for *TNFSF5, TNFRSF9, TNFRSF11a*, and *TNFRSF25 *were determined in a similar fashion, using commercially available gene expression assays (Hs00374176_m1, Hs00155512_m1, Hs00187189_m1, and Hs00237054_m1, respectively) and the 18S rRNA TaqMan^® ^Endogenous Control (Hs99999901_s1).

### Mutation screening

PCR amplifications of exons 1–6 were carried out using 100-μL reaction volumes with 1.5 mmol/L MgCl_2_; 200 μmol/L each of deoxy (d)-ATP, dGTP, dTTP, and dCTP; 2 pmol of each primer; 100 ng template DNA; and 1 U AmpliTaq Gold polymerase (Applied Biosystems, Foster City, CA). Amplifications of exon 7 were the same with the exception that the reaction mix had a concentration of 7% dimethylsulphoxide (DMSO). PCR cycling conditions were 10 min at 94°C, followed by 40 cycles of 94°C for 1 min, 65°C for 1 min, and 72°C for 1 min, followed by 15 min at 72°C. Sequencing reactions were setup using the BigDye Terminator Cycle Sequencing Reaction Kit with AmpliTaq DNA polymerase FS (Applied Biosystems, Foster City, CA) according to the manufacturer's specifications, and were subjected to gel electrophoresis on an ABI PRISM 3700 (Applied Biosystems, Foster City, CA). Sequencing data were aligned with the Sequencer program using *DFFB *sequence as reported by the Human Genome Database [14]. Forward and reverse PCR primer sequences are listed in Table 1.

**Table 1:** Primer sets used for amplifying and sequencing the coding regions of DFFB.

**Name**	**F primer seq**	**R primer seq**
DFFBamp1	gcttgcagagctcaccaggtgc	cggctgaggcgaacgaaaactacc
DFFBseq1	acggatctgagcagctgg	ctcctattctccccacacgc
DFFBamp2	aagcacagctcattccggtcg	tgatgggcacctggagctaagc
DFFBseq2	gccctcgtcttgagacc	aggacctcggagagtgc
DFFBamp3	gggggaagatgtggtcagaggctc	ccacctgagtccttgctgggtacc
DFFBseq3	cttgtgaccggggcag	atccaacttcttctggcacc
DFFBamp4	gctgtagtaagctgtgttcgtgccactg	gcgctagcttccctcaccagagc
DFFBseq4	ggaggacagagcaagacc	ccagatccacgcaagc
DFFBamp5	gggtctcagagggccatggag	cctgtgtgcactgcagcttgagag
DFFBseq5	atggatcgagagccagtg	ggcaagggctgaaggtc
DFFBamp6	cgggaggcggaggttgtagtaagc	ctgggctgtaacacgggtgcag
DFFBseq6	gccactgcactccagc	ccatggcagggacagg
DFFBamp7	gggaatttgtgaagagctgtgactgc	ccccaacaattcagaaatgtaatgaaatcag
DFFBseq7	gctatgacctgttgcctgtg	ggcacctgttaaaatgatgc

## Results and Discussions

**Figure 1:**
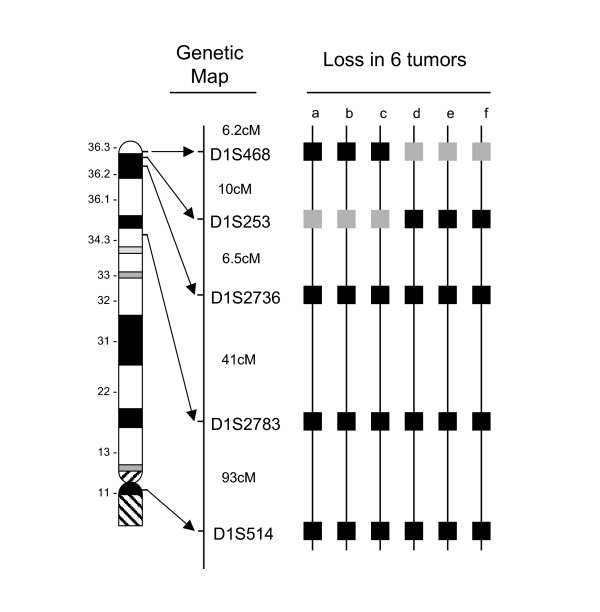
Common region of allelic loss on the short arm of chromosome 1 in oligodendrogliomas. Markers used for screening 1p-allelic loss and their placement on the genetic and cytogenetic maps of 1p. Black squares indicate where tumors retained allelic balance, whereas gray squares indicate allelic loss.

We evaluated 177 oligodendroglial tumors using QuMA for 1p-allelic loss in an attempt to determine a consensus region of deletion [11-13]. Loss was observed in 92 tumors, which in most cases involved the entire chromosomal arm. However, six tumors demonstrated localized loss involving the 1p36 region, defining a consensus region of deletion (Figure 1). These results were similar to those observed for other oligodendroglial 1p-deletion mapping studies, in which consensus regions of deletion involved 1p36 [5,6,8,9].

The second part of our strategy included the identification of abrogated cellular pathways in oligodendrogliomas with 1p/19q allelic loss. The transcriptsomes of eight pairs of gender- and age-matched tumors were measured using a Pathway microarray consisting of 1,500 functionally characterized genes constructed in our Cancer Genomics Core Laboratory. We used paired sample tests (the Sign Rank test and the paired t-test) to identify differentially expressed genes. While the sign rank test uses the null hypothesis that the medians for the two classes are same, the paired t-test uses the null hypothesis that the means of the two classes are same. We recognize a gene as significant when the sample data for the gene gives a p-value less than 0.01 (99% confidence). This analysis revealed a number of genes that demonstrated robust differential expression (McDonald and Zhang, unpublished results).

**Figure 2:**
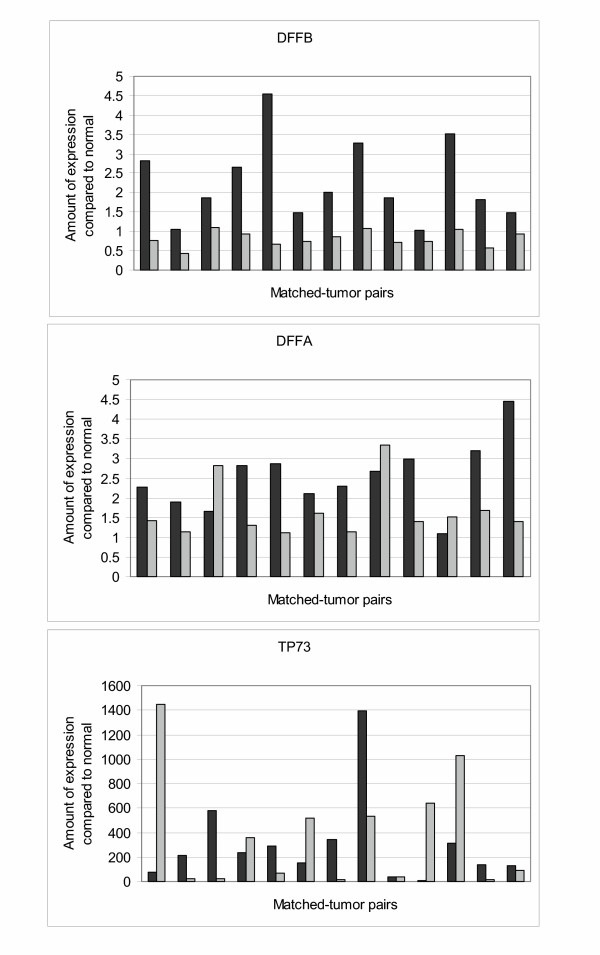
QRTPCR results in oligodendroglioma subsets. Black bar, 1p-intact samples; light gray bars, 1p-loss samples. A) Expression of *DFFB *was lower in all 1p-loss samples as compared to their matched 1p-intact samples. Ten of the thirteen 1p-loss tumors had lower expression of *DFFB *compared to normal brain, with three tumors demonstrating 1–2× the amount of normal brain *DFFB *expression. In contrast, all 1p-intact tumors had *DFFB *expression greater than or equal to that seen in normal brain. B) Expression of *DFFA *was lower in most 1p-loss samples as compared to their matched 1p-intact samples. Only three 1p-loss tumors had higher *DFFA *expression. Differential expression was detected to a degree; most 1p-loss tumors (10 of 12) demonstrated 1–2× the amount of normal brain *DFFA *expression, whereas most 1p-intact tumors (9of 12) demonstrated ≥ 2× the amount of normal brain *DFFA *expression. C). Differential *p73 *expression was not detected. In five of the pairs, 1p-loss tumors had higher expression, whereas in seven other pairs, 1p-intact tumors had higher expression.

We evaluated three of these genes with QRTPCR either because of location in our consensus region of deletion (*p73*), or due to their relationship to genes in our region of interest (*Tumor Necrosis Factor Super Family Ligand 5 *[*TNFSF5*] and *Tumor Necrosis Factor Receptor Super Family 11a *[*TNFRSF11a*]). Both *TNFSF5 *and *TNFRSF11a *are involved in apoptotic pathways that include several genes located in our region of interest: *TNFRSF9*, *TNFRSF25, DFFA*, and *DFFB*. Therefore, these genes were also tested via QRTPCR to determine if they had differential gene expression. Based on fresh/frozen tissue availability of the original 170 cases, total RNA samples from thirteen age- and gender-matched pairs of 1p/19q loss and intact tumors were evaluated for differential gene expression of *p73 *and the six TNF pathway genes. Of the seven genes, only *DFFB *was differentially expressed in all 13 pairs of tumor samples (Figure 2). Figure 2 also displays the differential expression levels for *DFFA *and *TP73 *for the tested tumor pairs. In contrast to *DFFB*, there were 3 pairs (25%) in which the 1p/19q loss tumors had higher *DFFA *expression. Likewise, 5 of the 13 pairs (38%) had higher *TP73 *expression in the 1p/19q loss tumors. Similarly, 33%, 50%, 50%, and 83% of tested pairs had higher expression of *TNFSF5*, *TNFRSF9*, *TNFRSF11a*, and *TNFRSF25 *in the 1p/19q loss tumors, respectively (data not shown). Since *DFFB *was the only tested gene that was differentially expressed in the same direction by all 13 pairs of tumors, we viewed *DFFB *as the best tumor suppressor gene candidate in our study.

We addressed the candidacy of *DFFB *as an oligodendroglioma tumor suppressor gene by mutation analysis. Twelve tumors with 1p-allelic loss were screened for mutations by sequencing the 1.2 kb coding region of *DFFB*. No coding region mutations were detected in any of the samples, which may indicate that haploinsufficiency of *DFFB *is enough of a genetic insult to contribute to tumorigenesis. In order to thoroughly test this hypothesis, it will be necessary to further investigate *DFFB*, perhaps by determining if the *DFFB *promoter has been hypermethylaed and/or if intronic sequence has been mutated in tumor samples.

*DFFB*-null mouse lines have been established via gene targeting [15]. Resultant mice developed normally but their lymphocytes were more susceptible to DNA damage. These animal model experiments suggest that *DFFB *is a weak tumor suppressor, which may only manifest its function in the presence of stress and DNA damage. Brain tissue samples from three six-month-old specimens revealed neuropil spongiosis, but no tumor development was observed (data not shown). We are not clear at present the implication of the neuropil spongiosis phenotype. Consistent with our data, a sequencing effort in a neuroblastoma study did not reveal a tumor-specific mutation in *DFFB *[16]. The gene expression level of *DFFB *was not analyzed in that study.

Thus, this study revealed that attenuated expression of the *DFFB *gene is a signature of oligodendrogliomas with 1p-allelic loss. Since *DFFB *contributes to both chromosomal condensation and DNA degradation during apoptosis, decreased expression of *DFFB *may subject cells to DNA damage stresses, which in turn may contribute to both tumorigenesis and better response to DNA damaging chemotherapy. Further studies are needed to investigate the role of the *DFFB *gene in the etiology of oligodendroglioma.
